# Identification of Novel Potential Vaccine Candidates against Tuberculosis Based on Reverse Vaccinology

**DOI:** 10.1155/2015/483150

**Published:** 2015-04-15

**Authors:** Gloria P. Monterrubio-López, Jorge A. González-Y-Merchand, Rosa María Ribas-Aparicio

**Affiliations:** Departamento de Microbiología, Escuela Nacional de Ciencias Biológicas (ENCB), Instituto Politécnico Nacional (IPN), Prolongación de Carpio y Plan de Ayala S/N, Colonia Santo Tomás, 11340 México, DF, Mexico

## Abstract

Tuberculosis (TB) is a chronic infectious disease, considered as the second leading cause of death worldwide, caused by *Mycobacterium tuberculosis*. The limited efficacy of the bacillus Calmette-Guérin (BCG) vaccine against pulmonary TB and the emergence of multidrug-resistant TB warrants the need for more efficacious vaccines. Reverse vaccinology uses the entire proteome of a pathogen to select the best vaccine antigens by *in silico* approaches. *M. tuberculosis* H37Rv proteome was analyzed with NERVE (New Enhanced Reverse Vaccinology Environment) prediction software to identify potential vaccine targets; these 331 proteins were further analyzed with VaxiJen for the determination of their antigenicity value. Only candidates with values ≥0.5 of antigenicity and 50% of adhesin probability and without homology with human proteins or transmembrane regions were selected, resulting in 73 antigens. These proteins were grouped by families in seven groups and analyzed by amino acid sequence alignments, selecting 16 representative proteins. For each candidate, a search of the literature and protein analysis with different bioinformatics tools, as well as a simulation of the immune response, was conducted. Finally, we selected six novel vaccine candidates, EsxL, PE26, PPE65, PE_PGRS49, PBP1, and Erp, from *M. tuberculosis* that can be used to improve or design new TB vaccines.

## 1. Introduction

Tuberculosis (TB) is a chronic infectious disease caused by an acid-fast bacillus,* Mycobacterium tuberculosis* [1]. TB is the second cause of death caused by an infectious agent throughout the world [2, 3]; in 2012, there were an estimated 8.6 million incident cases of TB globally, which is equivalent to 122 cases per 100,000 people, and the absolute number of cases continues to increase slightly from year to year [4].

The current vaccine against tuberculosis, bacillus Calmette-Guérin (BCG), exerts different levels of protection: from 46 to 100% against the disseminated disease form and from 0 to 80% against pulmonary disease [5, 6]. In addition to this low efficacy, reemergence of the disease caused by the appearance of the acquired immunodeficiency syndrome (AIDS) and multidrug-resistant (MDR) strains has generated requirements for a new and more efficient vaccine against TB [7].

The development of new vaccines starts with the identification of unique components of the microorganism capable of generating a protective immune response [3]. With traditional techniques, this could be a long and arduous process, aside from the difficulty of cultivating the microorganism in the laboratory [8–10].

Advances in sequencing technology and bioinformatics have resulted in an exponential growth of genome sequence information that has contributed to the development of software that aids genomic analysis in a short period of time and at a low cost. Reverse vaccinology (RV) applied to the genome of a pathogen aims to identify* in silico* the complete repertoire of immunogenic antigens that an organism is capable of expressing without the need of culturing the microorganism. Additionally, RV can help to discover novel antigens that might be less abundant, not expressed* in vitro*, or less immunogenic during infection that are likely to be missed by conventional approaches [8, 11–14].

The RV process begins with the proteomic information in a database; then, the selection of vaccine candidates is performed by means of different bioinformatics tools that analyze the properties of each protein and the human immune response generated by them [8–10, 15]. Good vaccine candidates are considered those that do not present homology with human proteins to avoid the generation of a potential autoimmune response [16]; these candidates must also lack transmembrane regions, in order to facilitate their expression. In addition, it is necessary to analyze the lack of cross-reaction among other pathogenic antigens [14]. Another characteristic a good vaccine candidate should have is to possess good antigenic and adhesin properties, which are important for the pathogenesis of the microorganism and for protection against the disease [13, 17]. Extracellular or cell surface localized proteins are good vaccine candidates due to their increased accessibility to the immune system [14, 16]. Currently, software useful for simulation of the immune response has been developed that could help in the search for novel vaccine candidates [15]. In this work, we have applied RV to the* M. tuberculosis* proteome with the purpose of selecting new antigens that could be used in a novel and more efficient vaccine against TB.

## 2. Materials and Methods

### 2.1. Proteome Analysis

New Enhanced Reverse Vaccinology (NERVE) software was downloaded, installed, and utilized to determine vaccine candidates employing the default parameters for Gram-positive bacteria [13]. The proteome sequences of* M. tuberculosis* H37Rv (NC_000962.2),* Mycobacterium bovis* AF2122/97 (NC_002945.3), and* M. bovis* BCG str. Pasteur 1173P2 (NC_008769.1) were downloaded from the Genome Project database of the National Center for Biotechnology Information (NCBI) [18]. Each proteome was analyzed individually by NERVE; conservation values for all proteins were determined comparing the* M. bovis* and BCG proteome against the* M. tuberculosis* proteome using the comparative option.

### 2.2. Antigenicity Determination

The antigenicity value was calculated for each protein using its amino acid sequences and the VaxiJen server, which predicts whether a protein could be a protective antigen. VaxiJen is based on auto cross covariance (ACC) and has a threshold of 0.5 in the antigenicity value [19].

### 2.3. Selection of Representative Proteins

With the parameters calculated with NERVE and VaxiJen, we selected proteins that presented an antigenicity value ≥0.5, 50% adhesin probability, and without homology with human proteins or transmembrane regions. The proteins selected were grouped according to the family of proteins to which they belong. In this manner, we obtained seven groups: ESX family proteins, PPE family proteins, PE family proteins, PE_PGRS family proteins, lipoproteins, hypothetic proteins, and, the last group, denominated “others,” composed of proteins with different miscellaneous characteristics. The amino acid sequence of each protein were downloaded from the NCBI protein database, and an alignment was made for each group of proteins using Clustal X software [20] in order to select representative proteins from each group.

### 2.4. Immune Response Simulation

With the amino acid sequences of the proteins selected, a human immune response simulation was performed using the C-ImmSim software to predict whether these proteins could generate a protective immune response against TB [15]. C-ImmSim simulates a portion of a lymph node but is not set up to simulate a realistic concentration of antigen; however, we adjusted the antigen concentration simulation to a high dose, comparable to a vaccination event. Different immunizations were simulated with each protein in the following two different schemes: first, a single immunization with each protein individually at time zero and, second, three immunizations at 0, 2, and 4 weeks with each protein separately. The level of Th1 cells stimulated 80 days after the first injection was identified.

### 2.5. Protein Analysis

The bioinformatics programs used to study the vaccine candidate's amino acid sequences included Phobius [21] to calculate and confirm protein subcellular localization more precisely, ANTHEPROT [22], Expasy [23], and IEDB software [24] and their different models for localizing protein regions with greater hydrophilic and greater solvent accessibility related with antigenic regions, and the SYFPEITHI ver. 1.0 program [25], which was used to determine the frequency of presentation of peptides to 35 different alleles of the major histocompatibility complex (MHC). In the case of lipoproteins, we employed only ProPred software [26] to determine the frequency of presentation of 25 amino acid peptides to different alleles of the MHC-II.

### 2.6. Bibliographic Study

Bibliographic information was sought for each protein using different databases on the website for information regarding its putative function, its use as vaccines, its role in virulence, its corresponding evaluated mutants, its induction of an immune response, and its level of conservation in mycobacterial proteomes.

### 2.7. Vaccine Candidate Selection

**Figure 1:**
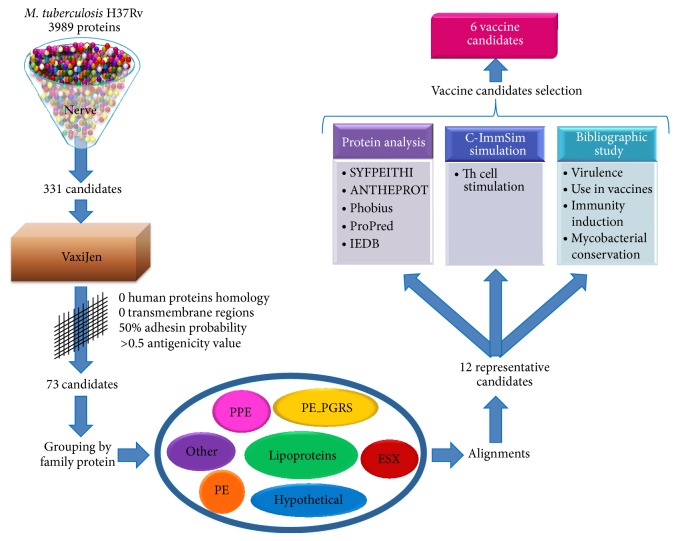
General workflow of the research. Reverse vaccinology was applied to the* M. tuberculosis* proteome to select novel vaccine candidates. The process starts with NERVE software selecting 331 vaccine candidates from 3989 proteins. These candidates were analyzed with different bioinformatics tools and bibliographic information selecting proteins representatives with the best values related with protective response. At the end of the study we chose six vaccine antigens (see details under Methods).

The vaccine candidates were selected using all the results, simulations, and bibliographic information obtained. The candidates possess the best values of the parameters calculated and exert diverse functions that render them useful as different targets in the microorganism (Figure 1).

## 3. Results and Discussion

### 3.1. Proteome Analysis

RV offers the advantage of reducing the time and cost of the development process of a new vaccine with the advantage of being safer and more effective. With the purpose of designing a new vaccine against TB with a greater protection level against pulmonary disease, we utilized RV to select vaccine candidates from the* M. tuberculosis* proteome.

The selection of potential vaccine candidates in this study was based on the analysis of several important properties [13, 19, 21, 27]. (1) Surface proteins or secreted proteins were selected because they are good targets of the immune system effector molecules. (2) Proteins with multiple transmembrane helices were discarded because they are not recommended for vaccine development, especially DNA vaccines, as they are difficult to clone, express, and purify. (3) Adhesin probability was considered an important factor since the first step in bacterial invasion is the contact with host molecules through adhesion structures, making adhesions good vaccine candidates capable of improving the immune response that results in blocking infection. (4) Proteins having similarity to those of the human proteome were avoided. The use of proteins or genes that encode them and having similitude with human proteins or DNA sequences can generate an autoimmune response or recombination and integration events in the host genome, respectively. (5) Proteins with the best values of antigenicity were chosen. Antigenicity is the property of the proteins to be recognized by the immune system; hence, it is desirable to find the highest antigenicity value for the selection of the best potential vaccine candidates. The* M. tuberculosis* proteome was studied with NERVE software, which identifies* in silico* vaccine candidates, analyzing the biological characteristics that influence vaccine design.

NERVE selected the* M. tuberculosis* H37Rv proteome, composed of 3989 proteins; the selection of candidates was performed considering the following characteristics: ≥50% adhesin probability, fewer than two transmembrane regions, and fewer than five proteins similar to the human proteome. In addition to this, the candidates must lack either membrane or cytoplasmic localization. Finally, NERVE selected 331 proteins as vaccine candidates (Additional file 1) (see Supplementary Material available online at http://dx.doi.org/10.1155/2015/483150).

The results were compared with the information deposited in the VIOLIN database [28], and we found several important matches in some antigens. Those coincidences provided support for the results obtained with NERVE. The vaccine candidates selected have diverse putative functions and different conservation values. Moreover, this software tool has the option of comparing the proteomes of two different organisms and of determining a conservation value among all the proteins. In this case, we compared the* M. tuberculosis* H37Rv proteome against the* M. bovis* and the BCG proteome [13, 14].

The proteome analysis was finalized by determining the antigenic value of the 331 vaccine candidates using the VaxiJen server to obtain protective antigens prediction.

### 3.2. Selection of Representative Proteins

Using the calculated characteristics, the number of vaccine candidates was reduced to 73 proteins, with a stricter selection, as mentioned in the Methods section. These proteins were grouped in seven clusters according to their type and the family to which they belong, as follows: the ESX protein family (3 proteins), the PPE protein family (7 proteins), the PE proteins family (8 proteins), PE_PGRS protein family (16 proteins), lipoproteins (5 proteins), hypothetical proteins (21 proteins), and the final group denominated “others” (13 proteins) (Table 1). This process was carried out because it is well known that members within the same protein family possess a close relationship between their sequences and functions.

With the purpose of selecting representative proteins from each group, we used amino acid sequences from their members to perform alignments using the Clustal X program, with the exception of those from the “others” and hypothetical proteins groups.

**Figure 2:**
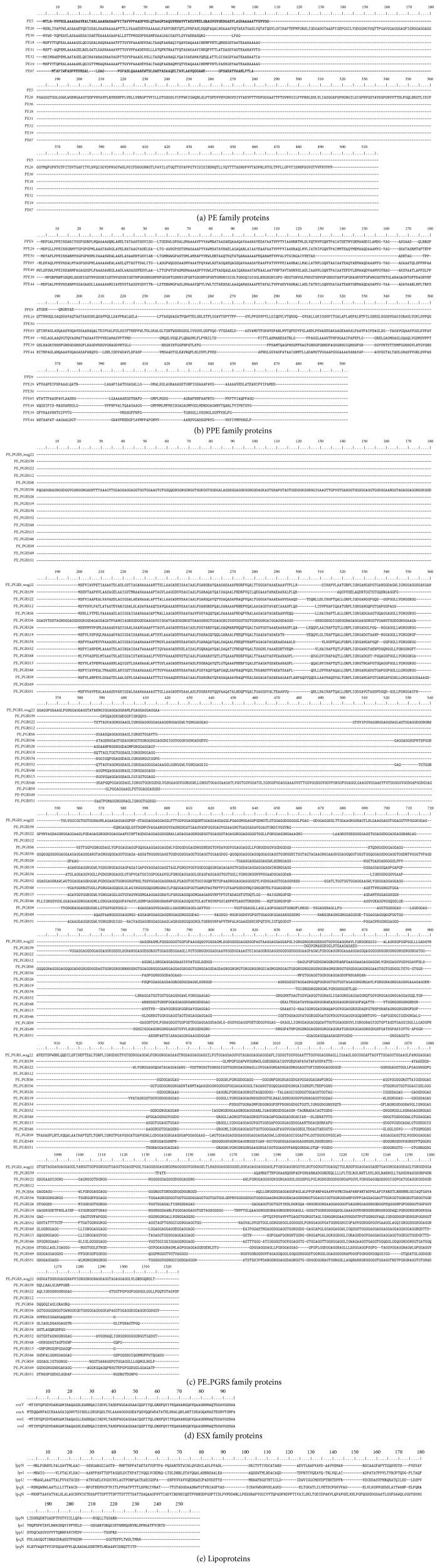
Amino acid sequence alignments using Clustal X for the vaccine candidates. The sequences are grouped by protein families and aligned using Clustal X software.

In the case of the hypothetic proteins, an alignment was carried out using the Psi-BLAST tool from the NCBI website, in order to grant them a putative function; in some cases, a coincidence was not found, but in others we could assume a probable function (Figure 2).

For the selection of representative proteins, we took into account the similarity among the sequences of the group, the best antigenicity values, the high probability to act as an adhesin, and their conservation in the* M. bovis* and BCG proteome. For this part, we chose 12 representative candidates from the seven protein families (Table 1).

### 3.3. Immune Response Simulation

**Figure 3:**
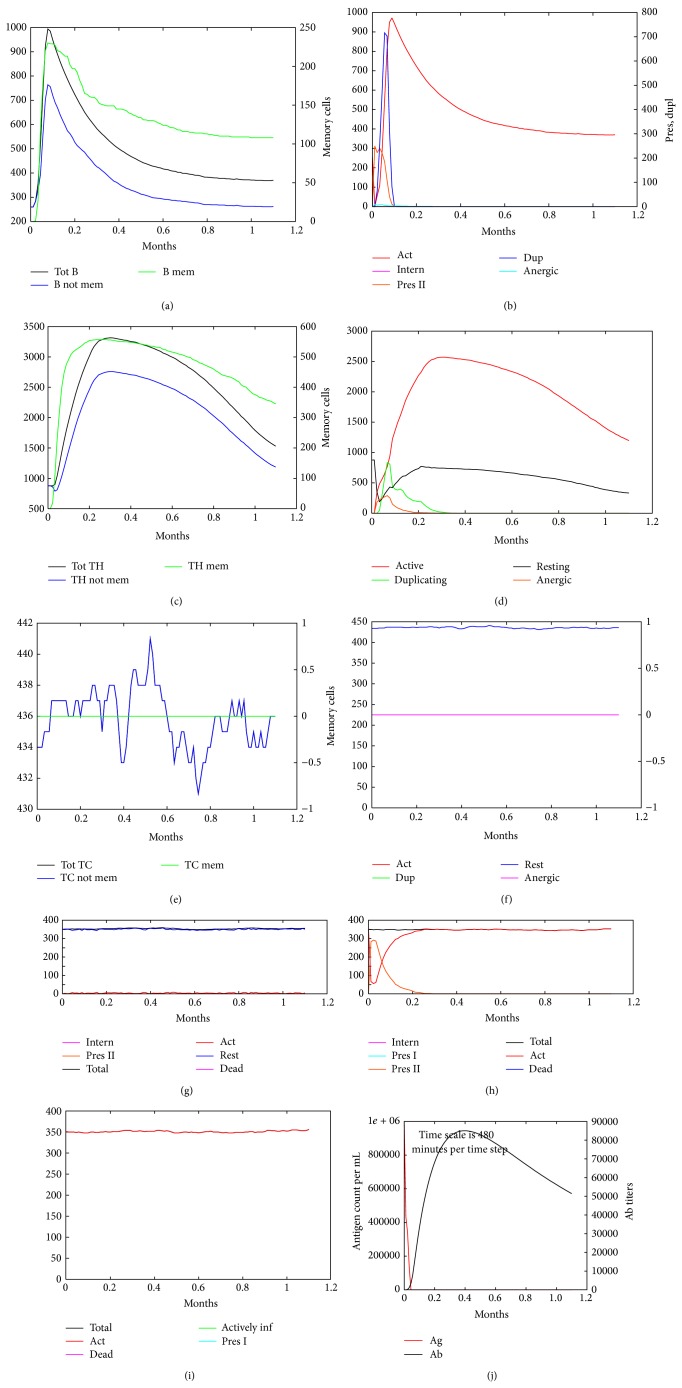
C-ImmSim simulation of an immunization experiment using Erp protein. An immunogenic molecule (Erp) was inoculated at time zero. Different cellular populations showed stimulation with Erp antigen at 1.2 months after one dose immunization. (a) B-cell population, (b) B-cell population per state, (c) Th cell population, (d) Th cell population per state, (e) Tc cell population, (f) Tc cell population per state, (g) macrophages population per state, (h) dendritic cell population per state, (i) epithelial cell population, and (j) antibody titers.

**Figure 4:**
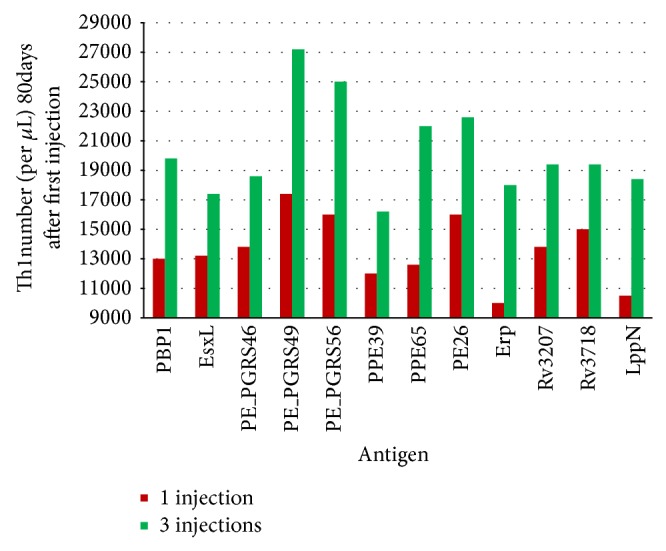
Levels of Th cells stimulated with vaccine candidates assessed with the C-ImmSim server. Simulation with C-ImmSim was performed for each vaccine candidate with one and three immunizations, and Th1 cells stimulated values per microliter were identified 80 days after the first immunization. The best stimulation was induced by PE_PGRS49 protein followed by PE_PGRS 56, PE26, PPE65, and PBP1 proteins.

The 12 proteins selected were used to conduct simulations of the human immune system response under different conditions with C-ImmSim software. In terms of the results of the simulations, C-ImmSim server showed the following nine graphs for each simulation: B-cell population, B-cell population per state, Th cell population, Th cell population per state, Tc population, Tc population per state, CD population, EP population, and Ab production (Figure 3). We found the same pattern in all the selected proteins with a slight difference among levels. However, we focused mainly on Th1 cells level including all states, because it has been reported that protective immunity against TB is conferred mainly by Th1 cells [14, 29]. We found that the levels of stimulated cells were most similar among the selected proteins when one immunization was performed, but this level improved when the number of immunizations increased, and there was also a remarkable differentiation among the proteins at the final simulation step. PE_PGRS family proteins showed the highest levels of stimulated Th cells, which also generated a good level of B cells stimulation, which is important for complementing the immune response (Figure 4).

### 3.4. Protein Analysis

The proteins were analyzed individually with several bioinformatics tools to determine whether the protein sequences had a region with important antigenic characteristics, that is, a region where there are matches with hydrophobicity, solvent accessibility, presentation to MHC, and antigenicity.

We did not find a protein that clearly possesses an antigenic region that could be used as an epitope or as a fusion in a vaccine. Conversely, we determined that all the proteins had high values of antigenicity in different parts of the sequence; thus, we recommend the use of complete proteins in a vaccine formulation because using only a fragment could eliminate some epitopes necessary for a complete and protective human immune response against the whole microorganism.

We also found that all of the proteins selected as vaccine candidates could be presented to several MHC with a high probability value, resulting in good probability of immune response induction against these components of* M. tuberculosis*.

In this analysis process, we identified protein subcellular localizations using Phobius tools to confirm the results emitted by NERVE, because Phobius software is more accurate than the program (HMMTOP) utilized by NERVE [21]. This characteristic is important in a vaccine candidate because proteins with cytoplasmic or membrane localization are less antigenic than extracytoplasmic proteins.

### 3.5. Bibliographic Study

We wanted to know whether the proteins would be safe if we used them on a vaccine formulation prior to the preclinical trials; thus, we studied the information published about different characteristics related with their impact on virulence.

We were able to observe that some proteins have not been studied, but we found information about other members of their family groups, such as PE, PPE, and PE_PGRS proteins, suggesting an influence on immune system evasion and antigenic variation, an important feature in considering a protein that will be included in a vaccine [39, 47, 59]; besides, PE_PGRS family proteins are restricted to pathogenic mycobacteria and, in particular, PE_PGRS11 and PE_PGRS 17 have been reported to induce maturation and activation of human dendritic cells, enhancing the ability of the latter to induce Th cells stimulation [26, 60].

In case of the antigen LppN we did not find specific information, besides, almost all the proteins in* M. tuberculosis* genome lack conserved regions, which means that they are unique proteins with different characteristics. Some lipoproteins are major antigens in the* Mycobacterium* genus that can generate also a cellular and humoral immune response but without immune memory response [50, 61–64].

Erp protein is a virulence factor present only in the* Mycobacterium* genus [51–53], it is an immunodominant antigen related to pathogenicity and is strongly induced in nutrient starvation related to the latency phase [53]. On the other hand, PBP1 protein is important in the replication phase because it catalyzes the final steps of bacterial cell wall peptidoglycan synthesis [54, 65, 66].

The EsxL candidate is an ESX-like protein with very similar characteristics to Esat-6, which is an immunodominant secreted protein used in research associated with the diagnosis of TB and new TB vaccines [30]. Esat-6 is a strong T-cell antigen, and its family members are involved in virulence and in host-pathogen interplay via either antigenic variation or antigenic drift [31, 32, 67].

We did not find any information related with hypothetical proteins Rv3207c and Rv3718c, and, up to date, it is unknown if these proteins could be of any risk within a vaccine formulation; thus further studies are needed to elucidate the role of these proteins in this kind of biological products. About the remainder candidates, we found no information that related them with high levels of bacterial virulence; conversely, we identified highlighted features that render these proteins as good vaccine candidates.

### 3.6. Vaccine Candidate Selection

All parameters studied were instrumental to determine the different and important characteristics to be taken into account for an adequate vaccine candidate. At the end of this study, we selected the following six proteins as the best vaccine candidates: EsxL (NCBI gene locus tag Rv1198), PE26 (NCBI gene locus tag Rv2519), PPE65 (NCBI gene locus tag Rv3621c), PE_PGRS49 (NCBI gene locus tag Rv3344c), PBP1 (NCBI gene locus tag Rv0050), and Erp (NCBI gene locus tag Rv3810). These proteins are representative of their family groups and have the best characteristics of antigenic value, adhesin probability, and stimulation of Th cells (Table 2). In addition, the information contained in the publications on these proteins indicates that they could be safe.

These proteins play different roles in mycobacterial pathogenicity and possess featured values of antigenicity and immune response induction; in addition, the PE_PGRS49 candidate is not present in* M. bovis* or in the BCG proteome, an important characteristic that could be useful in the improvement of a specific immune response against pulmonary disease in the current BCG vaccine.

## 4. Conclusions

The application of bioinformatics programs for the identification of proteins that could be used as vaccine candidates is a very useful, easier, and shorter process compared with traditional vaccinology, which is important for the research concerning public health.

Using RV, we selected six novel vaccine candidates from the* M. tuberculosis* H37Rv proteome, employing mainly* in silico* studies. The six proteins selected: EsxL, PE26, PPE65, PE_PGRS49, PBP1, and Erp correspond to several family proteins and possess different characteristics that are useful and important in vaccine design. The bibliographic information also indicated that they might be safe.

The potential vaccine candidates selected in this work could be used in different vaccine designs to conduct experiments in order to validate them as DNA vaccines, rBCG, or as recombinant proteins, to improve protection against TB, rendering the new vaccines more effective against the pulmonary disease.

We did not propose a specific and unique antigenic region within these protein structures; however, the bioinformatics and bibliographic analyses showed characteristics that make them valuable putative vaccine candidates that could be used further to experimentally investigate whether they are suitable for the development of a new vaccine against tuberculosis.

## Supplementary Material

Results emitted by NERVE after the *M. tuberculosis* H37Rv proteome analysis. NERVE selected 331 proteins out of 3989 proteins of *M. tuberculosis* H37Rv proteome. The selection of vaccine candidates was based on the analysis of several important properties: ≥50% of adhesion probability, fewer than two transmembrane regions, no protein similarity to human proteome, not membrane or cytoplasmic localization. Conserved values are also shown, comparing each candidate against *M. bovis* and BCG proteomes. Finally, NERVE search and obtain a putative function for each protein.

## Figures and Tables

**Table 1:** Proteins selected after reducing parameters and grouped in seven categories according their family group.

Protein family group	ID	Rv	VaxiJen antigenicity value	Length (amino acid)	Psi BLAST
ESX family	gi_15608177_ref_NP_215553_1_	Rv1037c	0.7444	94	ND
	**gi_15608338_ref_NP_215714_1_**	**Rv1198**	**0.6286**	**94**	**ND**
	gi_15610755_ref_NP_218136_1_	Rv3619c	0.7444	94	ND

PPE family	gi_57116729_ref_YP_177724_1_	Rv0388c (PPE9)	0.5334	180	ND
	gi_57116916_ref_YP_177840_1_	Rv1801 (PPE29)	0.5636	423	ND
	**gi_57116975_ref_YP_177871_1_**	**Rv2353c (PPE39)**	**0.897**	**354**	**ND**
	gi_57117024_ref_YP_177677_1_	Rv2770c (PPE44)	0.5056	382	ND
	gi_57117062_ref_YP_177932_1_	Rv3125c (PPE49)	0.5581	391	ND
	gi_57117064_ref_YP_177934_1_	Rv3135 (PPE50)	0.5029	132	ND
	**gi_57117135_ref_YP_177998_1_**	**Rv3621c (PPE65)**	**0.5241**	**413**	**ND**

PE family	gi_57116715_ref_YP_177710_1_	Rv0285 (PE5)	0.6696	102	ND
	gi_57116910_ref_YP_177834_1_	Rv1788 (PE18)	0.6228	99	ND
	gi_57116913_ref_YP_177837_1_	Rv1791 (PE19)	0.6125	94	ND
	**gi_57116998_ref_YP_177888_1_**	**Rv2519 (PE26)**	**0.718**	**492**	**ND**
	gi_57117110_ref_YP_177975_1_	Rv3477 (PE31)	0.5325	98	ND
	gi_57117136_ref_YP_177999_1_	Rv3622c (PE32)	0.5099	99	ND
	gi_57117151_ref_YP_178010_1_	Rv3739c (PE67)	0.5101	77	ND
	gi_57117167_ref_YP_178025_1_	Rv3893c (PE36)	0.5971	77	ND

PE_PGRS family	gi_57116752_ref_YP_177736_1_	Rv0532 (PE_PGRS6)	1.789	594	ND
	gi_57116773_ref_YP_177750_1_	Rv0746 (PE-PGRS9)	1.7153	783	ND
	gi_57116787_ref_YP_177759_1_	Rv0832 (PE_PGRS12)	0.6034	137	ND
	gi_57116793_ref_YP_177763_1_	Rv0872 (PE_PGRS15)	2.0866	606	ND
	gi_57116818_ref_YP_177780_1_	Rv1067c (PE_PGRS19)	2.2481	667	ND
	gi_57116826_ref_YP_177786_1_	Rv1091 (PE_PGRS22)	2.5016	853	ND
	gi_57116864_ref_YP_177811_1_	Rv1441c (PE_PGRS26)	2.1299	491	ND
	gi_57116905_ref_YP_177831_1_	Rv1795c (wag22)	2.0054	914	ND
	gi_57116924_ref_YP_177847_1_	Rv1840c (PE_PGRS34)	1.5912	515	ND
	gi_57116973_ref_YP_177869_1_	Rv2340c (PE_PGRS39)	1.0512	413	ND
	**gi_57117010_ref_YP_177896_1_**	**Rv2634c (PE_PGRS46)**	**2.146**	**778**	**ND**
	gi_57117029_ref_YP_177909_1_	Rv2853 (PE_PGRS48)	2.1046	615	ND
	**gi_57117093_ref_YP_177961_1_**	**Rv3344c (PE_PGRS49)**	**3.0927**	**484**	**ND**
	gi_57117098_ref_YP_177965_1_	Rv3367 (PE_PGRS51)	2.0713	588	ND
	gi_57117101_ref_YP_177968_1_	Rv3388 (PE_PGRS52)	2.2486	731	ND
	**gi_57117117_ref_YP_177981_1_**	**Rv3512 (PE_PGRS56)**	**3.4881**	**1079**	**ND**

Lipoproteins	gi_15607723_ref_NP_215097_1_	Rv0583c (LpqN)	0.6569	228	ND
	gi_15608368_ref_NP_215744_1_	Rv1228 (LpqX)	0.7609	185	ND
	gi_15608679_ref_NP_216057_1_	Rv1541c (LprI)	0.5298	197	ND
	**gi_15609407_ref_NP_216786_1_**	**Rv2270 (LppN)**	**0.5899**	**175**	**ND**
	gi_15609921_ref_NP_217300_1_	Rv2784c (LppU)	0.685	171	ND

Hypothetics	gi_15607199_ref_NP_214571_1_	Rv0057	0.6907	173	No matches
	gi_57116831_ref_YP_177639_1_	Rv1116A	0.6337	91	Related with PE family proteins of *Mycobacterium *
	gi_15608277_ref_NP_215653_1_	Rv1137c	0.76	122	No matches
	gi_15608941_ref_NP_216320_1_	Rv1804c	0.575	108	Related with* Mycobacterium* lipoproteins and gp53 *Mycobacterium* phage
	gi_15609051_ref_NP_216430_1_	Rv1914c	0.5202	135	No matches
	gi_15609215_ref_NP_216594_1_	Rv2078	0.5425	104	No matches
	gi_15609220_ref_NP_216599_1_	Rv2083	0.8189	314	No matches
	gi_15609401_ref_NP_216780_1_	Rv2264c	0.5862	592	Related with proline and threonine rich *Mycobacterium* proteins, like chaperone molecular and *Rhodococcus* hypothetical proteins
	gi_15609420_ref_NP_216799_1_	Rv2283	0.7336	64	No matches
	gi_15609429_ref_NP_216808_1_	Rv2292c	0.5396	74	No matches
	gi_15609439_ref_NP_216818_1_	Rv2302	0.958	80	Related with transduction signal protein and hypothetical proteins from *Nocardia* and *Frankia*, DNA binding protein from *Streptomyces *
	gi_15609797_ref_NP_217176_1_	Rv2660c	0.9073	75	No matches
	gi_15609843_ref_NP_217222_1_	Rv2706c	0.527	85	No matches
	gi_15610097_ref_NP_217476_1_	Rv2960c	0.6945	82	No matches
	gi_15610135_ref_NP_217514_1_	Rv2998	0.8339	153	No matches
	gi_15610204_ref_NP_217583_1_	Rv3067	0.566	136	No matches
	gi_15610316_ref_NP_217696_1_	Rv3180c	0.5182	144	Related with proteins which have pilT domain and with DNA binding proteins from *Mycobacterium *
	**gi_15610343_ref_NP_217723_1_**	**Rv3207c**	**0.6281**	**285**	**Related with membrane proteins and lipoproteins from *Corynebacterium* and *Streptomyces***
	gi_57117091_ref_NP_217854_2_	Rv3337	0.8824	128	Related with a putative hydrolase from *Mycobacterium *
	**gi_15610854_ref_NP_218235_1_**	**Rv3718c**	**0.7971**	**147**	**Related with KanY, polyketide cyclases, and lipid transport proteins from different species **
	gi_15611034_ref_NP_218415_1_	Rv3898c	0.7675	110	no matches

Others	gi_15607173_ref_NP_214545_1_	Rv0031	0.738	70	ND
	**gi_57116685_ref_YP_177687_1_**	**Rv0050 (PBP1)**	**0.6113**	**678**	**ND**
	gi_57116889_ref_NP_216091_2_	Rv1575	0.7415	166	ND
	gi_57117071_ref_YP_177941_1_	Rv3198A	0.6669	84	ND
	gi_15610488_ref_NP_217869_1_	Rv3352c	0.8742	123	ND
	**gi_15610946_ref_NP_218327_1_**	**Rv3810 (Erp)**	**0.6734**	**284**	**ND**
	gi_15609895_ref_NP_217274_1_	Rv2758c	0.6574	88	ND
	gi_57117060_ref_YP_177930_1_	Rv3118	0.833	100	ND
	gi_15610417_ref_NP_217798_1_	Rv3281	0.7311	177	ND
	gi_15611001_ref_NP_218382_1_	Rv3865	0.604	103	ND
	gi_15609778_ref_NP_217157_1_	Rv2641	0.7934	152	ND
	gi_15607954_ref_NP_215329_1_	Rv0814c	0.833	100	ND
	gi_57116926_ref_YP_177849_1_	Rv1860	0.5244	325	ND

Note: in bold are the highlighted representative proteins selected. ND: not determined.

**Table 2:** Vaccine candidates selected by reverse vaccinology.

Characteristic	ESXL	PE_PGRS49	PE26	PPE65	Erp	PBP1
Adhesin probability (%)	80	78	89	82	86	73

Antigenicity value (VaxiJen)	0.6286	3.0927	0.718	0.5241	0.6734	0.6113

Th1 cell number stimulated (1/3 injections)	13200/17400	17400/27200	16000/22600	12600/22000	10000/18000	13000/19800

*M. bovis* conservation	1.786	0	3.396	6.576	0.271	1.046

BCG conservation	1.786	0	3.298	6.656	0.271	1.046

Immunologic relation	It is suggested to be ESXA similar.	Their protein family members are important antigens recognized by vaccinated and TB patients serum. They are strongly related with antigenic drift and with evasion of immune response.	Their protein family members were used as strain differentiation markers, related with antigenic drift and with evasion of immune response and virulence.		Specific antibodies on cavitary TB patients have been detected.	Not reported

Characteristic or function	It is suggested to be ESXA similar.	The function of their members are varied, some of them are related with granuloma specific expression. They are also related with infectivity influence, necrosis, and apoptosis induction. They are restricted to *Mycobacterium* pathogenic species.	They are located on 3 conserved PAIs and 10% of the *Mycobacterium* genome is related with this family proteins.		Erp membrane protein precursor, phagosome produced. Their expression is related with nutrient reductions and *in vivo* after infection but before clinical tuberculosis in a rabbit model.	Penicillin binding protein of high molecular weight and membrane bound related with peptidoglycan synthesis. It is expressed only *in vivo*.

Vaccine trials	Not reported	A DNA vaccine with a protein related member Rv1818c has been reported, which generates protection only with the PE domain and better response with the complete protein.	Not reported		Not reported	Not reported

References	[30–38]	[29, 39–46]	[29, 32, 34, 47–49]		[26, 39, 50–52]	[53–58]

Note: ESXL (ESXL family); PE_PGRS49 (PE_PGRS family); PE26 (PE family); PPE65 (PPE family); Erp and PBP1 (Others).
